# Supplementary material for: Discovery and application of insertion-deletion (INDEL) polymorphisms for QTL mapping of early life-history traits in Atlantic salmon
Source: BMC Genomics. 2010 Mar 8;11:156. doi: 10.1186/1471-2164-11-156 (PMC2838853; doi:10.1186/1471-2164-11-156)
Supplement: Additional file 2 — Information on developed 76 locus single-run INDEL panel in Atlantic salmon. Information on fluorescence labeling, primer concentrations, PCR pooling and links to alignments, INDEL motifs and GENESCAN (Burge and Karlin 1997) predictions of genes/exons are available in html format. [file 1471-2164-11-156-S2.ZIP › Additionalfile2/table3060.html]

Contig Alignment

```
Base	A B C D E F G H I J K L M N O P Q R S   
1	G . . . . . . . . . . . . . . . . . .    
2	G . . . . . . . . . . . . . . . . . .    
3	G . . . . . . . . . . . . . . . . . .    
4	G . . . . . . . . . . . . . . . . . .    
5	T . . . . . . . . . . . . . . . . . .    
6	A . . . . . . . . . . . . . . . . . .    
7	C . . . . . . . . . . . . . . . . . .    
8	T . . . . . . . . . . . . . . . . . .    
9	A . . . . . . . . . . . . . . . . . .    
10	T . . . . . . . . . . . . . . . . . .    
11	C . . . . . . . . . . . . . . . . . .    
12	A . . . . . . . . . . . . . . . . . .    
13	C . . . . . . . . . . . . . . . . . .    
14	T . . . . . . . . . . . . . . . . . .    
15	G . . . . . . . . . . . . . . . . . .    
16	C . . . . . . . . . . . . . . . . . .    
17	T . . . . . . . . . . . . . . . . . .    
18	C . . . . . . . . . . . . . . . . . .    
19	A . . . . . . . . . . . . . . . . . .    
20	C . . . . . . . . . . . . . . . . . .    
21	A . . . . . . . . . . . . . . . . . .    
22	A . . . . . . . . . . . . . . . . . .    
23	A . . . . . . . . . . . . . . . . . .    
24	G . . . . . . . . . . . . . . . . . .    
25	C . . . . . . . . . . . . . . . . . .    
26	T . . . . . . . . . . . . . . . . . .    
27	T . . . . . . . . . . . . . . . . . .    
28	G . . . . . . . . . . . . . . . . . .    
29	T . . . . . . . . . . . . . . . . . .    
30	A . . . . . . . . . . . . . . . . . .    
31	G . . . . . . . . . . . . . . . . . .    
32	A . . . . . . . . . . . . . . . . . .    
33	G . . . . . . . . . . . . . . . . . .    
34	A . . . . . . . . . . . . . . . . . .    
35	T . . . . . . . . . . . . . . . . . .    
36	C . . . . . . . . . . . . . . . . . .    
37	C . . . . . . . . . . . . . . . . . .    
38	T . . . . . . . . . . . . . . . . . .    
39	G . . . . . . . . . . . . . . . . . .    
40	T . . . . . . . . . . . . . . . . . .    
41	T . . . . . . . . . . . . . . . . . .    
42	T . . . . . . . . . . . . . . . . . .    
43	C . . . . . . . . . . . . . . . . . .    
44	G . . . . . . . . . . . . . . . . . .    
45	T . . . . . . . . . . . . . . . . . .    
46	T . . . . . . . . . . . . . . . . . .    
47	T . . . . . . . . . . . . . . . . . .    
48	A . . . . . . . . . . . . . . . . . .    
49	C . . . . . . . . . . . . . . . . . .    
50	T . . . . . . . . . . . . . . . . . .    
51	T . . . . . . . . . . . . . . . . . .    
52	T . . . . . . . . . . . . . . . . . .    
53	C . . . . . . . . . . . . . . . . . .    
54	A . . . . . . . . . . . . . . . . . .    
55	C . . . . . . . . . . . . . . . . . .    
56	A . . . . . . . . . . . . . . . . . .    
57	G . . . . . . . . . . . . . . . . . .    
58	A . . . . . . . . . . . . . . . . . .    
59	T . . . . . . . . . . . . . . . . . .    
60	G . . . . . . . . . . . . . . . . . .    
61	G . . . . . . . . . . . . . . . . . .    
62	C . . . . . . . . . . . . . . . . . .    
63	T . . . . . . . . . . . . . . . . . .    
64	C . . . . . . . . . . . . . . . . . .    
65	C . . . . . . . . . . . . . . . . . .    
66	C . . . . . . . . . . . . . . . . . .    
67	C . . . . . . . . . . . . . . . . . .    
68	C . . . . . . . . . . . . . . . . . .    
69	C . . . . . . . . . . . . . . . . . .    
70	T . . . . . . . . . . . . . . . . . .    
71	G . . . . . . . . . . . . . . . . . .    
72	T . . . . . . . . . . . . . . . . . .    
73	G . . . . . . . . . . . . . . . . . .    
74	T . . . . . . . . . . . . . . . . . .    
75	C . . . . . . . . . . . . . . . . . .    
76	G . . . . . . . . . . . . . . . . . .    
77	A . . . . . . . . . . . . . . . . . .    
78	G . . . . . . . . . . . . . . . . . .    
79	A . . . . . . . . . . . . . . . . . .    
80	C . . . . . . . . . . . . . . . . . .    
81	A . . . . . . . . . . . . . . . . . .    
82	A . . . . . . . . . . . . . . . . . .    
83	G . . . . . . . . . . . . . . . . . .    
84	A . . . . . . . . . . . . . . . . . .    
85	C . . . . . . . . . . . . . . . . . .    
86	G . . . . . . . . . . . . . . . . . .    
87	A . . . . . . . . . . . . . . . . . .    
88	A . . . . . . . . . . . . . . . . . .    
89	G . . . . . . . . . . . . . . . . . .    
90	A . . . . . . . . . . . . . . . . . .    
91	A . . . . . . . . . . . . . . . . . .    
92	T . . . . . . . . . . . . . . . . . .    
93	C . . . . . . . . . . . . . . . . . .    
94	C . . . . . . . . . . . . . . . . . .    
95	G . . . . . . . . . . . . . . . . . .    
96	T . . . . . . . . . . . . . . . . . .    
97	T . . . . . . . . . . . . . . . . . .    
98	C . . . . . . . . . . . . . . . . . .    
99	A . . . . . . . . . . . . . . . . . .    
100	A . . . . . . . . . . . . . . . . . .    
101	G . . . . . . . . . . . . . . . . . .    
102	A . . . . . . . . . . . . . . . . . .    
103	G . . . . . . . . . . . . . . . . . .    
104	T . . . . . . . . . . . . . . . . . .    
105	T . . . . . . . . . . . . . . . . . .    
106	T . . . . . . . . . . . . . . . . . .    
107	G . . . . . . . . . . . . . . . . . .    
108	C . . . . . . . . . . . . . . . . . .    
109	C . . . . . . . . . . . . . . . . . .    
110	A . . . . . . . . . . . . . . . . . .    
111	A . . . . . . . . . . . . . . . . . .    
112	C . . . . . . . . . . . . . . . . . .    
113	A . . . . . . . . . . . . . . . . . .    
114	A . . . . . . . . . . . . . . . . . .    
115	A . . . . . . . . . . . . . . . . . .    
116	A . . . . . . . . . . . . . . . . . .    
117	T . . . . . . . . . . . . . . . . . .    
118	C . . . . . . . . . . . . . . . . . .    
119	C . . . . . . . . . . . . . . . . . .    
120	A . . . . . . . . . . . . . . . . . .    
121	A . . . . . . . . . . . . . . . . . .    
122	G . . . . . . . . . . . . . . . . . .    
123	G . . . . . . . . . . . . . . . . . .    
124	G . . . . . . . . . . . . . . . . . .    
125	C . . . . . . . . . . . . . . . . . .    
126	T . . . . . . . . . . . . . . . . . .    
127	C . . . . . . . . . . . . . . . . . .    
128	C . . . . . . . . . . . . . . . . . .    
129	T . . . . . . . . . . . . . . . . . .    
130	A A . . . . . . . . . . . . . . . . .    
131	G G . . . . . . . . . . . . . . . . .    
132	C C . . . . . . . . . . . . . . . . .    
133	C C . . . . . . . . . . . . . . . . .    
134	A A . . . . . . . . . . . . . . . . .    
135	C C . . . . . . . . . . . . . . . . .    
136	C C . . . . . . . . . . . . . . . . .    
137	G G . . . . . . . . . . . . . . . . .    
138	A A . . . . . . . . . . . . . . . . .    
139	G G . . . . . . . . . . . . . . . . .    
140	C C . . . . . . . . . . . . . . . . .    
141	T T . . . . . . . . . . . . . . . . .    
142	C C . . . . . . . . . . . . . . . . .    
143	G G . . . . . . . . . . . . . . . . .    
144	G G . . . . . . . . . . . . . . . . .    
145	C C . . . . . . . . . . . . . . . . .    
146	G G . . . . . . . . . . . . . . . . .    
147	T T . . . . . . . . . . . . . . . . .    
148	G G . . . . . . . . . . . . . . . . .    
149	G G . . . . . . . . . . . . . . . . .    
150	T T . . . . . . . . . . . . . . . . .    
151	C C . . . . . . . . . . . . . . . . .    
152	T T . . . . . . . . . . . . . . . . .    
153	C C . . . . . . . . . . . . . . . . .    
154	A A . . . . . . . . . . . . . . . . .    
155	A A . . . . . . . . . . . . . . . . .    
156	C C . . . . . . . . . . . . . . . . .    
157	A A . . . . . . . . . . . . . . . . .    
158	C C . . . . . . . . . . . . . . . . .    
159	A A . . . . . . . . . . . . . . . . .    
160	G G . . . . . . . . . . . . . . . . .    
161	A A . . . . . . . . . . . . . . . . .    
162	T T . . . . . . . . . . . . . . . . .    
163	T T . . . . . . . . . . . . . . . . .    
164	A A . . . . . . . . . . . . . . . . .    
165	C C C . . . . . . . . . . . . . . . .    
166	C C C . . . . . . . . . . . . . . . .    
167	A A A . . . . . . . . . . . . . . . .    
168	A A A . . . . . . . . . . . . . . . .    
169	A A A . . . . . . . . . . . . . . . .    
170	G G G . . . . . . . . . . . . . . . .    
171	C C C . . . . . . . . . . . . . . . .    
172	A A A . . . . . . . . . . . . . . . .    
173	G G G . . . . . . . . . . . . . . . .    
174	A A A . . . . . . . . . . . . . . . .    
175	C C C . . . . . . . . . . . . . . . .    
176	A A A . . . . . . . . . . . . . . . .    
177	A A A . . . . . . . . . . . . . . . .    
178	A A A . . . . . . . . . . . . . . . .    
179	G G G . . . . . . . . . . . . . . . .    
180	C C C . . . . . . . . . . . . . . . .    
181	C C C . . . . . . . . . . . . . . . .    
182	G G G . . . . . . . . . . . . . . . .    
183	A A A . . . . . . . . . . . . . . . .    
184	G G G . . . . . . . . . . . . . . . .    
185	C C C . . . . . . . . . . . . . . . .    
186	A A A . . . . . . . . . . . . . . . .    
187	C C C . . . . . . . . . . . . . . . .    
188	G G G . . . . . . . . . . . . . . . .    
189	T T T . . . . . . . . . . . . . . . .    
190	G G G . . . . . . . . . . . . . . . .    
191	A A A . . . . . . . . . . . . . . . .    
192	A A A . . . . . . . . . . . . . . . .    
193	A A A . . . . . . . . . . . . . . . .    
194	A A A . . . . . . . . . . . . . . . .    
195	G G G . . . . . . . . . . . . . . . .    
196	G G G . . . . . . . . . . . . . . . .    
197	A A A . . . . . . . . . . . . . . . .    
198	A A A . . . . . . . . . . . . . . . .    
199	A A A . . . . . . . . . . . . . . . .    
200	C C C . . . . . . . . . . . . . . . .    
201	G G G . . . . . . . . . . . . . . . .    
202	G G G . . . . . . . . . . . . . . . .    
203	C C C . . . . . . . . . . . . . . . .    
204	A A A . . . . . . . . . . . . . . . .    
205	C C C . . . . . . . . . . . . . . . .    
206	G G G . . . . . . . . . . . . . . . .    
207	T T T . . . . . . . . . . . . . . . .    
208	T T T . . . . . . . . . . . . . . . .    
209	G G G . . . . . . . . . . . . . . . .    
210	C C C . . . . . . . . . . . . . . . .    
211	A A A . . . . . . . . . . . . . . . .    
212	C C C . . . . . . . . . . . . . . . .    
213	C C C . . . . . . . . . . . . . . . .    
214	A A A . . . . . . . . . . . . . . . .    
215	C C C . . . . . . . . . . . . . . . .    
216	A A A . . . . . . . . . . . . . . . .    
217	C C C . . . . . . . . . . . . . . . .    
218	A A A . . . . . . . . . . . . . . . .    
219	C C C . . . . . . . . . . . . . . . .    
220	T T T . . . . . . . . . . . . . . . .    
221	A A A . . . . . . . . . . . . . . . .    
222	C C C . . . . . . . . . . . . . . . .    
223	A A A . . . . . . . . . . . . . . . .    
224	A A A . . . . . . . . . . . . . . . .    
225	C C C . . . . . . . . . . . . . . . .    
226	C C C . . . . . . . . . . . . . . . .    
227	T T T . . . . . . . . . . . . . . . .    
228	C C C . . . . . . . . . . . . . . . .    
229	A A A . . . . . . . . . . . . . . . .    
230	A A A . . . . . . . . . . . . . . . .    
231	A A A . . . . . . . . . . . . . . . .    
232	T T T . . . . . . . . . . . . . . . .    
233	T T T . . . . . . . . . . . . . . . .    
234	T T T . . . . . . . . . . . . . . . .    
235	G G G . . . . . . . . . . . . . . . .    
236	G G G . . . . . . . . . . . . . . . .    
237	G G G . . . . . . . . . . . . . . . .    
238	T T T . . . . . . . . . . . . . . . .    
239	G G G . . . . . . . . . . . . . . . .    
240	C C C . . . . . . . . . . . . . . . .    
241	C C C . . . . . . . . . . . . . . . .    
242	A A A . . . . . . . . . . . . . . . .    
243	G G G . . . . . . . . . . . . . . . .    
244	A A A . . . . . . . . . . . . . . . .    
245	C C C . . . . . . . . . . . . . . . .    
246	C C C . . . . . . . . . . . . . . . .    
247	A A A . . . . . . . . . . . . . . . .    
248	C C C . . . . . . . . . . . . . . . .    
249	C C C . . . . . . . . . . . . . . . .    
250	T T T . . . . . . . . . . . . . . . .    
251	C C C . . . . . . . . . . . . . . . .    
252	C C C . . . . . . . . . . . . . . . .    
253	T T T . . . . . . . . . . . . . . . .    
254	A A A . . . . . . . . . . . . . . . .    
255	T T T . . . . . . . . . . . . . . . .    
256	G G G . . . . . . . . . . . . . . . .    
257	G G G . . . . . . . . . . . . . . . .    
258	G G G . . . . . . . . . . . . . . . .    
259	C C C . . . . . . . . . . . . . . . .    
260	T T T . . . . . . . . . . . . . . . .    
261	T T T . . . . . . . . . . . . . . . .    
262	C C C . . . . . . . . . . . . . . . .    
263	A A A . . . . . . . . . . . . . . . .    
264	T T T . . . . . . . . . . . . . . . .    
265	G G G . . . . . . . . . . . . . . . .    
266	G G G . . . . . . . . . . . . . . . .    
267	T T T . . . . . . . . . . . . . . . .    
268	C C C . . . . . . . . . . . . . . . .    
269	C C C . . . . . . . . . . . . . . . .    
270	G G G . . . . . . . . . . . . . . . .    
271	C C C . . . . . . . . . . . . . . . .    
272	A A A . . . . . . . . . . . . . . . .    
273	G G G . . . . . . . . . . . . . . . .    
274	C C C . . . . . . . . . . . . . . . .    
275	T T T . . . . . . . . . . . . . . . .    
276	C C C . . . . . . . . . . . . . . . .    
277	T T T . . . . . . . . . . . . . . . .    
278	G G G . . . . . . . . . . . . . . . .    
279	G G G . . . . . . . . . . . . . . . .    
280	G G G . . . . . . . . . . . . . . . .    
281	G G G . . . . . . . . . . . . . . . .    
282	T T T . . . . . . . . . . . . . . . .    
283	G G G . . . . . . . . . . . . . . . .    
284	G G G . . . . . . . . . . . . . . . .    
285	A A A . . . . . . . . . . . . . . . .    
286	G G G . . . . . . . . . . . . . . . .    
287	G G G . . . . . . . . . . . . . . . .    
288	A A A . . . . . . . . . . . . . . . .    
289	G G G . . . . . . . . . . . . . . . .    
290	C C C . . . . . . . . . . . . . . . .    
291	T T T . . . . . . . . . . . . . . . .    
292	C C C . . . . . . . . . . . . . . . .    
293	A A A . . . . . . . . . . . . . . . .    
294	G G G . . . . . . . . . . . . . . . .    
295	G G G . . . . . . . . G . . . . . . .    
296	A A A . . . . . . . . A . . . . . . .    
297	T T T . . . . . . . . T . . . . . . .    
298	C C C . . . . . . . . C . . . . . . .    
299	A A A . . . . . . . . A . . . . . . .    
300	T G G . . . . . . . . T . . . . . . .    
301	C C C . . . . . . . . C . . . . . . .    
302	A A A . . . . . . . . A . A . . . . .    
303	G G G . . . . . . . . G . G . . . . .    
304	G G G . . . . . . . . G . G . . . . .    
305	A A A . . . . . . . . A . A . . . . .    
306	T T T . . . . . . . . T . T . . . . .    
307	G G G . . . . . . . . G . G . . . . .    
308	G G G . . . . . . . . G . G . . . . .    
309	C C C . . . . . . . . C . C . . . . .    
310	C C C . . . . . . . . C . C . . . . .    
311	C C C . . . . . . . . C . C . . . . .    
312	A A A . . . . . . . . A . A . . . . .    
313	G G G . . . . . . . . G . G . . . . .    
314	C C C . . . . . . . . C . C . . . . .    
315	A A A . . . . . . . . A . A . . . . .    
316	G G G . . . . . . . . G . G . . . . .    
317	G G G . . . . . . . . G . G . . . . .    
318	T T T . . . . . . . . T . T . . . . .    
319	G G G . . . . . . . . G . G . . . . .    
320	A A A . . . . . . . . A . A . . . . .    
321	A A A . . . . . . . . A . A . . . . .    
322	G G G . . . . . . . . G . G . . . . .    
323	G G G . . . . . . . . G . G . . . . .    
324	A A A . . . . . . . . A . A . . . . .    
325	G G G . . . . . . . . G . G . . . . .    
326	G G G . . . . . . . . G . G . . . . .    
327	T T T . . . . . . . . T . T . . . . .    
328	T T T . . . . . . . . T . T . . . . .    
329	C C C . . . . . . . . C . C . . . . .    
330	T T T . . . . . . . . T . T . . . . .    
331	G G G . . . . . . . . G . G . . . . .    
332	C C C . . . . . . . . C . C . . . . .    
333	C C C . . . . . . . . C . C . . . . .    
334	T T T T . . . . . . . T . T . . . . .    
335	G G G G . . . . . . . G . G . . . . .    
336	A A A A . . . . . . . A . A . . . . .    
337	C C C C . . . . . . . C . C . . . . .    
338	A A A A . . . . . . . A . A . . . . .    
339	T T T T . . . . . . . T . T . . . . .    
340	C C C C . . . . . . . C . C . . . . .    
341	C C C C . . . . . . . C . C . . . . .    
342	C C C C . . . . . . . C . C . . . . .    
343	C C C C . . . . . . . C . C . . . . .    
344	C C C C . . . . . . . C . C . . . . .    
345	T T T T . . . . . . . T . T T . . . .    
346	C C C C . . . . . . . C . C C . . . .    
347	G G G G . . . . . . . G . G G . . . .    
348	G G G G . . . . . . . G . G G . . . .    
349	C C C C . . . . . . . C . C C . . . .    
350	G G G G . . . . . . . G . G G . . . .    
351	T T T T . . . . . . . T . T T . . . .    
352	C C C C . . . . . . . C . C C . . . .    
353	A A A A . . . . . . . A . A A . . . .    
354	T T T T . . . . . . . T . T T . . . .    
355	C C C C . . . . . . . C . C C . . . .    
356	A A A A . . . A . . . A . A A . . . .    
357	C C C C . . . C . . . C . C C . . . .    
358	C C C C . . . C . . . C . C C . . . .    
359	A A A A . . . A . . . A . A A . . . .    
360	G G G G . . . G . . . G . G G . . . .    
361	A A A A . . . A . . . A . A A . . . .    
362	G G G G . . . G . . . G . G G . . . .    
363	A A A A . . . A . . . A . A A . . . .    
364	C C C C . . . C . . . C . C C . . . .    
365	C C C C . . . C . . . C . C C . . . .    
366	T T T T . . . T . . . T . T T . . . .    
367	G G G G . . . G . . . G . G G . . . .    
368	G G G G . . . G . . . G . G G . . . .    
369	T T T T . . . T . . . T . T T . . . .    
370	G G G G . G . G . . . G . G G . . . .    
371	C C C C . C . C . . . C . C C . . . .    
372	A A A A . A . A . . . A . A A . . . .    
373	A A A A . A . A . . . A . A A . . . A    
374	A A A A . A . A . . . A . A A . . . A    
375	C C C C . C . C . . . C . C C . . . C    
376	C C C C . C . C . . . C . C C . . . C    
377	A A A A . A . A . . . A . A A . . . A    
378	A A A A . A . A . . . A A A A . . . A    
379	C C C C . C . C . . . C C C C . . . C    
380	T T T T . T . T . . . T T T T . . . T    
381	G G G G . G . G . . . G G G G . . . G    
382	C C C C . C . C . . . C C C C . . . C    
383	G G G G . G . G . . . G G G G . . . G    
384	T T T T . T . T . . . T T T T . . . T    
385	G G G G . G . G . . . G G G G . . . G    
386	G G G G . G . G . . . G G G G . . . G    
387	A A A A . A . A . . . A A A A . . . A    
388	T T T T . T . T . . . T T T T . . . T    
389	A A A A . A . A . . . A A A A . . . A    
390	C C C C . C . C . . . C C C C . . . C    
391	C C C C . C . C . . . C C C C . . . C    
392	A A A A . A . A . . . A A A A . . . A    
393	C C C C . C . C . . . C C C C . . . C    
394	T T T T . T . T . . . T T T T . . . T    
395	A A A A . A . A . . . A A A A . . . A    
396	T T T T . T . T . . . T T T T . . . T    
397	C C C C . C . C . . . C C C C . . . C    
398	A A A A . A A A . . . A A A A . . . A    
399	C C C C . C C C . . . C C C C . . . C    
400	T T T T . T T T . . . T T T T . . . T    
401	A A A A . A A A . . . A A A A . . . A    
402	A A A A . A A A . . . A A A A . . . A    
403	C C C C . C C C . . . C C C C . . . C    
404	C C C C . C C C . . . C C C C . . . C    
405	T T T T . T T T . . . T T T T . . . T    
406	G G G G . G G G . . . G G G G . . . G    
407	C C C C . C C C . . . C C C C . . . C    
408	T T T T . T T T . . . T T T T . . . T    
409	G G G G . G G G . . . G G G G . . . G    
410	G G G G . G G G . . . G G G G . . . G    
411	A A A A . A A A . . . A A A A . . . A    
412	G G G G . G G G . . . G G G G . . . G    
413	A A A A . A A A . . . A A A A . . . A    
414	G G G G . G G G . . . G G G G . . . G    
415	C C C C . C C C . . . C C C C . . . C    
416	A A A A . A A A . . . A A A A . . . A    
417	C C C C . C C C . . . C C C C . . . C    
418	T T T T . T T T . . . T T T T . . . T    
419	G G G G . G G G . . . G G G G . G . G    
420	A A A A . A A A . . . A A A A . A . A    
421	G G G G . G G G . . . G G G G . G . G    
422	G G G G . G G G . . . G G G G . G . G    
423	A A A A . A A A . . . A A A A . A . A    
424	G G G G . G G G . . . G G G G . G . G    
425	T T T T . T T T . . . T T T T . T . T    
426	T T T T . T T T . . . T T T T . T . T    
427	T T T T . T T T . . . T T T T . T . T    
428	C C C C . C C C . . . C C C C . C . C    
429	C C C C . C C C . . . C C C C . C . C    
430	A A A A . A A A . . . A A A A . A . A    
431	T T T T . T T T . . . T T T T . T . T    
432	T T T T . T T T . . . T T T T . T . T    
433	G G G G . G G G . . . G G G G . G . G    
434	G G G G . G G G . . . G G G G . G . G    
435	A A A A . A A A . . . A A A A . A . A    
436	G G G G . G G G . . . G G G G . G . G    
437	G G G G . G G G . . . G G G G . G . G    
438	C C C C . C C C . . . C C C C . C . C    
439	C C C C . C C C . . . C C C C . C . C    
440	A A A A . A A A . . . A A A A . A . A    
441	C C C C . C C C . . . C C C C . C . C    
442	A A A A . A A A . . . A A A A . A . A    
443	G G G G . G G G . . . G G G G . G . G    
444	G G G G . G G G . . . G G G G . G . G    
445	C C C C . C C C . . . C C C C . C . C    
446	G G G G . G G G . . . G G G G . G . G    
447	G G G G . G G G . . . G G G G . G . G    
448	A A A A . A A A . . . A A A A . A . A    
449	G G G G . G G G . . . G G G G . G . G    
450	C C C C . C C C . . . C C C C . C . C    
451	C C C C . C C C . . . C C C C . C . C    
452	A A A A . A A A . . . A A A A . A . A    
453	C C C C . C C C . . . C C C C . C . C    
454	A A A A . A A A . . . A A A A . A . A    
455	T T T T . T T T . . . T T T T . T . T    
456	C C C C . C C C . . . C C C C . C . C    
457	T T T T . T T T . . . T T T T . T . T    
458	G G G G . G G G . . . G G G G G G . G    
459	C C C C . C C C . . . C C C C C C . C    
460	A A A A . A A A . . . A A A A A A . A    
461	C C C C . C C C . . . C C C C C C . C    
462	C C C C . C C C . . . C C C C C C . C    
463	A A A A . A A A . . . A A A A A A . A    
464	G G G G . G G G . . . G G G G G G . G    
465	G G G G . G G G . . . G G G G G G . G    
466	C C C C . C C C . . . C C C C C C . C    
467	C C C C . C C C . . . C C C C C C . C    
468	C C C C . C C C . . . C C C C C C . C    
469	A A A A . A A A . . . A A A A A A . A    
470	T T T T . T T T . . . T T T T T T . T    
471	C C C C . C C C . . . C C C C C C . C    
472	C C C C . C C C . . . C C C C C C . C    
473	T T T T . T T T . . . T T T T T T . T    
474	G G G G . G G G . . . G G G G G G . G    
475	G G G G . G G G . . . G G G G G G . G    
476	A A A A . A A A . . . A A A A A A . A    
477	G G G G . G G G . . . G G G G G G . G    
478	G G G G . G G G . . . G G G G G G . G    
479	T T T T . T T T . . . T T T T T T . T    
480	C C C C . C C C . . . C C C C C C . C    
481	C C C C . C C C . . . C C C C C C . C    
482	C C C C . C C C . . . C C C C C C . C    
483	T T T T . T T T . . . T T T T T T . T    
484	C C C C . C C C . . . C C C C C C . C    
485	T T T T . T T T . . . T T T T T T . T    
486	C C C C . C C C . . . C C C C C C . C    
487	C C C C . C C C . . . C C C C C C . C    
488	G G G G . G G G . . . G G G G G G . G    
489	T T T T . T T T . . . T T T T T T . T    
490	T T T T . T T T . . . T T T T T T . T    
491	T T T T . T T T . . . T T T T T T . T    
492	C C C C . C C C . . . C C C C C C . C    
493	A A A A . A A A . . . A A A A A A . A    
494	G G G G . G G G . . . G G G G G G . G    
495	C C C C . C C C . . . C C C C C C . C    
496	T T T T . T T T . . . T T T T T T . T    
497	A A A A . A A A . . . A A A A A A . A    
498	C C C C . C C C . . . C C C C C C . C    
499	A A A A . A A A . . . A A A A A A . A    
500	C C C C . C C C . . . C C C C C C . C    
501	C C C C . C C C . . . C C C C C C . C    
502	T T T T . T T T . . . T T T T T T . T    
503	G G G G . G G G . . . G G G G G G . G    
504	C C C C . C C C . . . C C C C C C . C    
505	T T T T T T T T . . . T T T T T T . T    
506	C C C C C C C C . . . C C C C C C . C    
507	C C C C C C C C . . . C C C C C C . C    
508	C C C C C C C C . . . C C C C C C . C    
509	A A A A A A A A . . . A A A A A A . A    
510	C C C C C C C C . . . C C C C C C . C    
511	C C C C C C C C . . . C C C C C C . C    
512	A A A A A A A A . . . A A A A A A . A    
513	T T T T T T T T . . . T T T T T T . T    
514	T T T T T T T T . . . T T T T T T . T    
515	A A A A A A A A . . . A A A A A A . A    
516	A A A A A A A A . . . A A A A A A . A    
517	G G G G G G G G . . . G G G G G G . G    
518	C C C C C C C C . . . C C C C C C . C    
519	C C C C C C C C . . . C C C C C C . C    
520	T T T T T T T T . . . T T T T T T . T    
521	G G G G G G G G . . . G G G G G G . G    
522	C C C C C C C C . . . C C C C C C . C    
523	T T T T T T T T . . . T T T T T T . T    
524	G G G G G G G G . . . G G G G G G . G    
525	C C C C C C C C . . . C C C C C C . C    
526	C C C C C C C C . . . C C C C C C . C    
527	A A A A A A A A . . . A A A A A A . A    
528	A A A A A A A A . . . A A A A A A . A    
529	G G G G G G G G . . . G G G G G G . G    
530	T T T T T T T T . . . T T T T T T . T    
531	C C C C C C C C . . . C C C C C C . C    
532	T T T T T T T T . . . T T T T T T . T    
533	T T T T T T T T . . . T T T T T T . T    
534	T T T T T T T T . . . T T T T T T . T    
535	T T T T T T T T . . . T T T T T T . T    
536	G G G G G G G G . . . G G G G G G . G    
537	G G G G G G G G . . . G G G G G G . G    
538	G G G G G G G G . . . G G G G G G . G    
539	A A A A A A A A . . . A A A A A A . A    
540	A A A A A A A A . . . A A A A A A . A    
541	A A A A A A A A . . . A A A A A A . A    
542	T T T T T T T T . . . T T T T T T . T    
543	C C C C C C C C . . . C C C C C C . C    
544	G G G G G G G G . . . G G G G G G . G    
545	C C C C C C C C . . . C C C C C C . C    
546	C C C C C C C C . . . C C C C C C . C    
547	A A A A A A A A . . . A A A A A A . A    
548	G G G G G G G G . . . G G G G G G . G    
549	T T T T T T T T . . . T T T T T T . T    
550	A A A A A A A A . . . A A A A A A . A    
551	G G G G G G G G . . . G G G G G G . G    
552	A A A A A A A A . . . A A A A A A . A    
553	C C C C C C C C . . . C C C C C C . C    
554	C C C C C C C C . . . C C C C C C . C    
555	G G G G G G G G . . . G G G G G G . G    
556	A A A A A A A A . . . A A A A A A . A    
557	C C C C C C C C . . . C C C C C C . C    
558	A A A A A A A A . . . A A A A A A . A    
559	C C C C C C C C . . . C C C C C C . C    
560	A A A A A A A A . . . A A A A A A . A    
561	T T T T T T T T . . . T T T T T T . T    
562	G G G G G G G G G G . G G G G G G . G    
563	T T T T T T T T T T . T T T T T T . T    
564	C C C C C C C C C C . C C C C C C . C    
565	C C C C C C C C C C . C C C C C C . C    
566	C C C C C C C C C C . C C C C C C . C    
567	T T T T T T T T T T . T T T T T T . T    
568	A A A A A A A A A A . A A A A A A . A    
569	C C C C C C C C C C . C C C C C C . C    
570	A A A A A A A A A A . A A A A A A . A    
571	A A A A A A A A A A . A A A A A A . A    
572	G G G G G G G G G G . G G G G G G . G    
573	A A A A A A A A A A . A A A A A A . A    
574	G G G G C G G G G G . G G G G G G . G    
575	A A A A A A A A A A . A A A A A A . A    
576	G G G G G G G G G G . G G G G G G . G    
577	G G G G G G G G G G . G G G G G G . G    
578	A A A A A A A A A A . A A A A A A . A    
579	A A A A A A A A A A . A A A A A A . A    
580	G G G G G G G G G G . G G G G G G . G    
581	G G G G G G G G G G . G G G G G G . G    
582	A A A A A A A A A A . A A A A A A . A    
583	G G G G G G G G G G . G G G G G G . G    
584	G G G G G G G G G G . G G G G G G . G    
585	C C C C C C C T C C . C T C C C C . C    
586	C C C C C C C C C C . C C C C C C . C    
587	A A A A A A A A A A . A A A A A A . A    
588	T T T T T T T T T T . T T T T T T . T    
589	G G G G G G G G G G . G G G G G G . G    
590	T T T T T T T T T T . T T T T T T . T    
591	A A A A A A A A A A . A A A A A A . A    
592	T T T T T T T T T T . T T T T T T . T    
593	G G G G G G G G G G . G G G G G G . G    
594	A A A A A A A A A A . A A A A A A . A    
595	G G G G G G G G G G . G G G G G G . G    
596	T T T T T T T T T T . T T T T T T . T    
597	T T T T T T T T T T . T T T T T T . T    
598	T T T T T T T T T T . T T T T T T . T    
599	G G G G G G G G G G . G G G G G G . G    
600	C C C C C C C C C C . C C C C C C . C    
601	A A A A A A A A A A . A A A A A A . A    
602	A A A A A A A A A A . A A A A A A . A    
603	G G G G G G G G G G . G G G G G G . G    
604	A A A A A A A A A A . A A A A A A . A    
605	A A A A A A A A A A . A A A A A A . A    
606	G G G G G G G G G G . G G G G G G . G    
607	A A A A A A A A A A . A A A A A A . A    
608	C C C C C C C C C C . C C C C C C . C    
609	G G G G G G G G G G . G G G G G G . G    
610	C C C C C C C C C C . C C C C C C . C    
611	T T T T T T T T T T . T T T T T T . T    
612	A A A A A A A A A A . A A A A A A . A    
613	C C C C C C C C C C . C C C C C C . C    
614	A A A A A A A A A A . A A A A A A . A    
615	T T T T T T T T T T . T T T T T T . T    
616	C C C C C C C C C C . C C C C C C . C    
617	G G G G G G G G G G . G G G G G G . G    
618	A A A A A A A A A A . A A A A A A . A    
619	G G G G G G G G G G . G G G G G G . G    
620	A A A A A A A A A A . A A A A A A . A    
621	A A A A A A A A A A . A A A A A A . A    
622	G G G G G G G G G G . G G G G G G . G    
623	C C C C C C C C C C . C C C C C C . C    
624	A A A A A A A A A A . A A A A A A . A    
625	T T T T T T T T T T . T T T T T T . T    
626	G G G G G G G G G G . G G G G G G . G    
627	G G G G G G G G G G . G G G G G G . G    
628	A A A A A A A A A A . A A A A A A . A    
629	C C C C C C C C C C . C C C C C C . C    
630	T T T T T T T T T T . T T T T T T . T    
631	G G G G G G G G G G . G G G G G G . G    
632	G G G G G G G G G G . G G G G G G . G    
633	A A A A A A A A A A . A A A A A A . A    
634	G G G G G G G G G G . G G G G G G . G    
635	C C C C C C C C C C . C C C C C C . C    
636	A A A A A A A A A A . A A A A A A . A    
637	G G G G G G G G G G . G G G G G G . G    
638	G G G G G G G G G G . G G G G G G . G    
639	A A A A A A A A A A . A A A A A A . A    
640	C C C C C C C C C C . C C C C C C . C    
641	G G G G G G G G G G . G G G G G G . G    
642	A A A A A A A A A A . A A A A A A . A    
643	C C C C C C C C C C . C C C C C C . C    
644	A A A A A A A A A A . A A A A A A . A    
645	A A A A A A A A A A . A A A A A A . A    
646	C C C C C C C C C C . C C C C C C . C    
647	C C C C C C C C C C . C C C C C C . C    
648	T T T T T T T T T T . T T T T T T . T    
649	C C C C C C C C C C . C C C C C C . C    
650	T T T T T T T T T T . T T T T T T . T    
651	G G G G G G G G G G . G G G G G G . G    
652	A A A A A A A A A A . A A A A A A . A    
653	A A A A A A A A A A . A A A A A A . A    
654	C C C C C C C C C C . C C C C C C . C    
655	A A A A A A A A A A . A A A A A A . A    
656	A A A A A A A A A A . A A A A A A . A    
657	G G G G G G G G G G . G G G G G G . G    
658	A A A A A A A A A A . A A A A A A . A    
659	T T T T T T T T T T . T T T T T T . T    
660	C C C C C C C C C C . C C C C C C . C    
661	T T T T T T T T T T . T T T T T T . T    
662	G G G G G G G G G G . G G G G G G . G    
663	A A A A A A A A A A . A A A A A A . A    
664	T T T T T T T T T T . T T T T T T . T    
665	C C C C C C C C C C . C C C C C C . C    
666	G G G G G G G G G G . G G G G G G . G    
667	C C C C C C C C C C . C C C C C C . C    
668	T T T T T T T T T T . T T T T T T . T    
669	G G G G G G G G G G . G G G G G G . G    
670	G G G G G G G G G G . G G G G G G . G    
671	G G G G G G G G G G . G G G G G G . G    
672	A A A A A A A A A A . A A A A A A . A    
673	A A A A A A A A A A . A A A A A A . A    
674	A A A A A A A A A A . A A A A A A . A    
675	T T T T T T T T T T . T T T T T T . T    
676	G G G G G G G G G G . G G G G G G . G    
677	T T T T T T T T T T . T T T T T T . T    
678	G G G G G G G G G G . G G G G G G . G    
679	T T T T T T T T T T . T T T T T T . T    
680	T T T T T T T T T T . T T T T T T . T    
681	A A A A A A A A A A A A A A A A A A A    
682	G G G G G G G G G G G G G G G G G G G    
683	G G G G G G G G G G G G G G G G G G G    
684	A A A A A A A A A A A A A A A A A A A    
685	A A A A A A A A A A A A A A A A A A A    
686	T T T T T T T T T T T T T T T T T T T    
687	G G G G G G G G G G G G G G G G G G G    
688	A A A A A A A A A A A A A A A A A A A    
689	T T T T T T T T T T T T T T T T T T T    
690	G G G G G G G G G G G G G G G G G G G    
691	T T T T T T T T T T T T T T T T T T T    
692	T T T T T T T T T T T T T T T T T T T    
693	T T T T T T T T T T T T T T T T T T T    
694	G G G G G G G G G G G G G G G G G G G    
695	T T T T T T T T T T T T T T T T T T T    
696	T T T T T T T T T T T T T T T T T T T    
697	A A A A A A A A A A A A A A A A A A A    
698	A A A A A A A A A A A A A A A A A A A    
699	A A A A A A A A A A A A A A A A A A A    
700	A A A A A A A A A A A A A A A A A A A    
701	T T T T T T T T T T T T T T T T T T T    
702	C C C C C C C C C C C C C C C C C C C    
703	A A A A A A A A A A A A A A A A A A A    
704	A A A A A A A A A A A A A A A A A A A    
705	A A A A A A A A A A A A A A A A A A A    
706	T T T T T T T T T T T T T T T T T T T    
707	A A A A A A A A A A A A A A A A A A A    
708	A A A A A A A A A A A A A A A A A A A    
709	T T T T T T T T T T T T T T T T T T T    
710	T T T T T T T T T T T T T T T T T T T    
711	G G G G G G G G G G G G G G G G G G G    
712	T T T T T T T T T T T T T T T T T T T    
713	A A A A A A A A A A A A A A A A A A A    
714	C C C C C C C C C C C C C C C C C C C    
715	A A A A A A A A A A A A A A A A A A A    
716	G G G G G G G G G G G G G G G G G G G    
717	A A A A A A A A A A A A A A A A A A A    
718	C C C C C C C C C C C C C C C C C C C    
719	A A A A A A A A A A A A A A A A A A A    
720	A A A A A A A A A A A A A A A A A A A    
721	T T T T T T T T T T T T T T T T T T T    
722	A A A A A A A A A A A A A A A A A A A    
723	A A A A A A A A A A A A A A A A A A A    
724	T T T T T T T T T T T T T T T T T T T    
725	T T T T T T T T T T T T T T T T T T T    
726	G G G G G G G G G G G G G G G G G G G    
727	T T T T T T T T T T T T T T T T T T T    
728	A A A A A A A A A A A A A A A A A A A    
729	C C C C C C C C C C C C C C C C C C C    
730	A A A A A A A A A A A A A A A A A A A    
731	A A A A A A A A A A A A A A A A A A A    
732	G G G G G G G G G G G G G G G G G G G    
733	A A A A A A A A A A A A A A A A A A A    
734	G G G G G G G G G G G G G G G G G G G    
735	T T T T T T T T T T T T T T T T T T T    
736	G G G G G G G G G G G G G G G G G G G    
737	T T T T T T T T T T T T T T T T T T T    
738	A A A A A A A A A A A A A A A A A A A    
739	T T T T T T T T T T T T T T T T T T T    
740	G G G G G G G G G G G G G G G G G G G    
741	G G G G G G G G G G G G G G G G G G G    
742	A A A A A A A A A A A A A A A A A A A    
743	G G G G G G G G G G G G G G G G G G G    
744	A A A A A A A A A A A A A A A A A A A    
745	G G G G G G G G G G G G G G G G G G G    
746	T T T T T T T T T T T T T T T T T T T    
747	T T T T T T T T T T T T T T T T T T T    
748	G G G G G G G G G G G G G G G G G G G    
749	A A A A A A A A A A A A A A A A A A A    
750	A A A A A A G A A A A A A A A A A A A    
751	C C C C C C C C C C C C C C C C C C C    
752	A A A A A A A A A A A A A A A A A A A    
753	G G G G G G G G G G G G G G G G G G G    
754	A A A A A A A A A A A A A A A A A A A    
755	G G G G G G G G G G G G G G G G G G G    
756	G G G G G G G G G G G G G G G G G G G    
757	G G G G G G G G G G G G G G G G G G G    
758	T T T T T T T T T T T T T T T T T T T    
759	T T T T T T T T T T T T T T T T T T T    
760	T T T T T T T T T T T T T T T T T T T    
761	T T T T T T T T T T T T T T T T T T T    
762	. T T T T T T T T T T T T T T T T T T    
763	. G G G G G G G G G G G G G G G G G G    
764	. T T T T T T T T T T T T T T T T T T    
765	. T T T T T T T T T T T T T T T T T T    
766	. G G G G G G G G G G G G G G G G G G    
767	. A A A A A A A A A A A A A A A A A A    
768	. G G G G G G G G G G G G G G G G G G    
769	. C C C C C C C C C C C C C C C C C C    
770	. C C C C C C C C C C C C C C C C C C    
771	. A A A A A A A A A A A A A A A A A A    
772	. C C C C C C C C C C C C C C C C C C    
773	. A A A A A A A A A A A A A A A A A A    
774	. C C C C C C C C C C C C C C C C C C    
775	. G G G G G G G G G G G G G G G G G G    
776	. T T T T T T T T T T T T T T T T T T    
777	. A A A A A A A A A A A A A A A A A A    
778	. C C C C C C C C C C C C C C C C C C    
779	. T T T T T T T T T T T T C C T T T T    
780	. G G G G G G G G G G G G G G G G G G    
781	. G G G G G G G G G G G G G G G G G G    
782	. A A A A A A A A A A A A A A A A A A    
783	. G G G G G G G G G G G G G G G G G G    
784	. G G G G G G G G G G G G G G G G G G    
785	. A A A A A A A A A A A A A A A A A A    
786	. A A A A A A A A A A A A A A A A A A    
787	. A A A A A A A A A A A A A A A A A A    
788	. A A A A A A A A A A A A A A A A A A    
789	. C C C C C C C C C C C C C C C C C C    
790	. C C C C C C C C C C T C C C C C C C    
791	. T T T T T T T T T T T T T T T T T T    
792	. T T T T T T T T T T T T T T T T T T    
793	. C C C C C C C C C C C C C C C C C C    
794	. T T T T T T T T T T T T T T T T T T    
795	. G G G G G G G G G G G G G G G G G G    
796	. A A A A A A A A A A A A A A A A A A    
797	. A A A A A A A A A A A A A A A A A A    
798	. G G G G G G G G G G G G G G G G G G    
799	. A A A A A A A A A A A A A A A A A A    
800	. C C C C C C C C C C C C C C C C C C    
801	. T T T T T T T T T T T T T T T T T T    
802	. T T T T T T T T T T T T T T T T T T    
803	. C C C C C C C C C C C C C C C C C C    
804	. A A A A A A A A A A A A A A A A A A    
805	. A A A A A A A A A A A A A A A A A A    
806	. T T T T T T T T T T T T T T T T T T    
807	. G G G G G G G G G G G G G G G G G G    
808	. G G G G G G G G G G G G G G G G G G    
809	. A A A A A A A A A A A A A A A A A A    
810	. T T T T T T T T T T T T T T T T T T    
811	. T T T T T T T T T T T T T T T T T T    
812	. T T T T T T T T T T T T T T T T T T    
813	. T T T T T T T T T T T T T T T T T T    
814	. C C C C C C C C C C C C C C C C C C    
815	. T T T T T T T T T T T T T T T T T T    
816	. T T T T T T T T T T T T T T T T T T    
817	. T T T T T T T T T T T T T T T T T T    
818	. A A A A A A A A A A A A A A A A A A    
819	. T T T T T T T T T T T T T T T T T T    
820	. T T T T T T T T T T T T T T T T T T    
821	. T T T T T T T T T T T T T T T T T T    
822	. T T T T T T T T T T T T T T T T T T    
823	. T T T T T T T T T T T T T T T T T T    
824	. T T T T T T T T T T T T T T T T T T    
825	. A A A A A A A A A A A A A A A A A A    
826	. T T T T T T T T T T T T T T T T T T    
827	. T T T T T T T T T T T T T T T T T T    
828	. T T T T T T T T T T T T T T T T T T    
829	. G G G G G G G G G G G G G G G G G G    
830	. C C C C C C C C C C C C C C C C C C    
831	. T T T T T T T T T T T T T T T T T T    
832	. T T T T T T T T T T T T T T T T T T    
833	. G G G G G G G G G G G G G G G G G G    
834	. C C C C C C C C C C C C C C C C C C    
835	. A A A A A A A A A A A A A A A A A A    
836	. T T T T T T T T T T T T T T T T T T    
837	. G G G G G G G G G G G G G G G G G G    
838	. G G G G G G G G G G G G G G G G G G    
839	. A A A A A A A A A A A A A A A A A A    
840	. A A A A A A A A A A A A A A A A A A    
841	. A A A A A A A A A A A A A A A A A A    
842	. T T T T T T T T T T T T T T T T T T    
843	. C C C C C C C C C C C C C C C C C C    
844	. A A A A A A A A A A A A A A A A A A    
845	. T T T T T T T T T T T T T T T T T T    
846	. G G G G G G G G G G G G G G G G G G    
847	. C C C C C C C C C C C C C C C C C C    
848	. A A A A A A A A A A A A A A A A A A    
849	. T T T T T T T T T T T T T T T T T T    
850	. A A G G G G G G G G G G G G G G G G    
851	. T T T T G T T T T T T T T T T T T T    
852	. G G G G G G G G G G G G G G G G G G    
853	. T T T T T T T T T T T T T T T T T T    
854	. A A A A A A A A A A A A A A A A A A    
855	. T T T T T T T T T T T T T T T T T T    
856	. T T T T T T T T T T T T T T T T T T    
857	. T T T T T T T T T T T T T T T T T T    
858	. T T T T T T T T T T T T T T T T T T    
859	. T T T T T T T T T T T T T T T T T T    
860	. A A A A A A A A A A A A A A A A A A    
861	. A A A A A A A A A A A A A A A A A A    
862	. T T T T T T T T T T T T T T T T T T    
863	. T T T T T T T T T T T T T T T T T T    
864	. C C C C C C C C C C C C C C C C C C    
865	. T T T T T T T T T T T T T T T T T T    
866	. T T T T T T T T T T T T T T T T T T    
867	. T T T T T T T T T T T T T T T T T T    
868	. C C C C C C C C C C C C C C C C C C    
869	. T T T T T T T T T T T T T T T T T T    
870	. C C C C C C C C C C C C C C C C C C    
871	. C C C C C C C C C C C C C C C C C C    
872	. C C C C C C C C C C C C C C C C C C    
873	. A A A A A A A A A A A A A A A A A A    
874	. G G G G G G G G G G G G G G G G G G    
875	. C C C C C C C C C C C C C C C C C C    
876	. A A A A A A A A A A A A A A A A A A    
877	. A A A A A A A A A A A A A A A A A A    
878	. A A A A A A A A A A A A A A A A A A    
879	. A A A A A A A A A A A A A A A A A A    
880	. A A A A A A A A A A A A A A A A A A    
881	. T T T T T T T T T T T T T T T T T T    
882	. A A A A A A A A A A A A A A A A A A    
883	. T T T T T T T T T T T T T T T T T T    
884	. T T T T T T T T T T T T T T T T T T    
885	. A A A A A A A A A A A A A A A A A A    
886	. T T T T T T T T T T T T T T T T T T    
887	. T T T T T T T T T T T T T T T T T T    
888	. T T T T T T T T T T T T T T T T T T    
889	. T T T T T T T T T T T T T T T T T T    
890	. G G G G G G G G G G G G G G G G G G    
891	. A A A A A A A A A A A A A A A A A A    
892	. T T T T T T T T T T T T T T T T T T    
893	. A A A A A A A A A A A A A A A A A A    
894	. A A A A A A A A A A A A A A A A A A    
895	. C C C C C C C C C C C C C C C C C C    
896	. A A A A A A A A A A A A A A A A A A    
897	. A A A A A A A A A A A A A A A A A A    
898	. T T T T T T T T T T T T T T T T T T    
899	. T T T T T T T T T T T T T T T T T T    
900	. T T T T T T T T T T T T T T T T T T    
901	. G G G G G G G G G G G G G G G G G G    
902	. T T T T T T T T T T T T T T T T T T    
903	. A A A A A A A A A A A A A A A A A A    
904	. T T T T T T T T T T T T T T T T T T    
905	. T T T T T T T T T T T T T T T T T T    
906	. A A A A A A A A A A A A A A A A A A    
907	. A A A A A A A A A A A A A A A A A A    
908	. G G G G G G G G G G G G G G G G G G    
909	. A A A A A A A A A A A A A A A A A A    
910	. C C C C C C C C C C C C C C C C C C    
911	. C C C C C C C C C C C C C C C C C C    
912	. C C C C C C C C C C C C C C C C C C    
913	. A A A A A A A A A G A A A A A A G A    
914	. G G G G G G G G G G G G G G G G G G    
915	. T T T T T T T T T T T T T T T T T T    
916	. G G G G G G G G G G G G G G G G G G    
917	. A A A A A A A A A A A A A A A A A A    
918	. C C C C C C C C C C C C C C C C C C    
919	. C C C C C C C C C C C C C C C C C C    
920	. . . A A A A A A A A A A A A A A A A    
921	. . . T T T T T T T T T T T T T T T T    
922	. . . A . A A A A A A A A A A A A A A    
923	. . . A . A A A A A A A A A A A A A A    
924	. . . A . A A A A A A A A A A A A A A    
925	. . . A . A A A A A A A A A A A A A A    
926	. . . C . C C C C C C C C C C C C C C    
927	. . . A . A A A A A A A A A A A A A A    
928	. . . T . T T T T T T T T T T T T T T    
929	. . . C . C C C C C C C C C C C C C C    
930	. . . T . T T T T T T T T T T T T T T    
931	. . . C . C C C C C C C C C C C C C C    
932	. . . A . A A A A A A A A A A A A A A    
933	. . . T . T T T T T T T T T T T T T T    
934	. . . T . T T T T T T T T T T T T T T    
935	. . . C . C C C C C C C C C C C C C C    
936	. . . G . G G G G G G G G G G G G G G    
937	. . . T . T T T T T T T T T T T T T T    
938	. . . T . T T T T T T T T T T T T T T    
939	. . . . . T T T T T T T T T T T T T T    
940	. . . . . T T T T T T T T T T T T T T    
941	. . . . . T T T T T T T T T T T T T T    
942	. . . . . A A A A A A A A A A A A A A    
943	. . . . . T T T T T T T T T T T T T T    
944	. . . . . A A A A A A A A A A A A A A    
945	. . . . . A A A A A A A A A A A A A A    
946	. . . . . T T T T T T T T T T T T T T    
947	. . . . . G G G G G G G G G G G G G G    
948	. . . . . G G G G G G G G G G G G G G    
949	. . . . . C C C C C C C C C C C C C C    
950	. . . . . C C C C C C C C C C C C C C    
951	. . . . . G G G G G G G G G G G G G G    
952	. . . . . A A A A A A A A A A A A A A    
953	. . . . . G G G G G G G G G G G G G G    
954	. . . . . G G G G G G G G G G G G G G    
955	. . . . . T T T T T T T T T T T T T T    
956	. . . . . T T T T T T T T T T T T T T    
957	. . . . . A A A A A A A A A A A A A A    
958	. . . . . T T T T T T T T T T T T T T    
959	. . . . . G G G G G G G G G G G G G G    
960	. . . . . A A A A A A A A A A A A A A    
961	. . . . . T T T T T T T T T T T T T T    
962	. . . . . A A A A A A A A A A A A A A    
963	. . . . . A A A A A A A A A A A A A A    
964	. . . . . C C C C C C C C C C C C C C    
965	. . . . . A A A A A A A A A A A A A A    
966	. . . . . - C - C C C C - - - - C C -  7	 indel  
967	. . . . . - A - A A A A - - - - A A -  7	 indel  
968	. . . . . A A A A A A A A A A A A A A    
969	. . . . . A A A A A A A A A A A A A A    
970	. . . . . G G G G G G G G G G G G G G    
971	. . . . . T T T T T T T T T T T T T T    
972	. . . . . C C C C C C C C C C C C C C    
973	. . . . . C C C C C C C C C C C C C C    
974	. . . . . A A A A A A A A A A A A A A    
975	. . . . . T T C T T T T C T T T T T T    
976	. . . . . C C C C C C C C C C C C C C    
977	. . . . . T T T T T T T T T T T T T T    
978	. . . . . T T T T T T T T T T T T T T    
979	. . . . . G G G G G G G G G G G G G G    
980	. . . . . C C C C C C C C C C C C C C    
981	. . . . . T T T T T T T T T T T T T T    
982	. . . . . C C C C C C C C C C C C C C    
983	. . . . . T T T T T T T T T T T T T T    
984	. . . . . G G G G G G G G G G G G G G    
985	. . . . . C C C C C C C C C C C C C C    
986	. . . . . T T T T T T T T T T T T T T    
987	. . . . . T T T T T T T T T T T T T T    
988	. . . . . T T T T T T T T T T T T T T    
989	. . . . . C C C C C C C C C C C C C C    
990	. . . . . A A A A A A A A A A A A A A    
991	. . . . . G G G G G G G G G G G G G G    
992	. . . . . A A A A A A A A A A A A A A    
993	. . . . . G G G G G G G G G G G G G G    
994	. . . . . A A A A A A A A A A A A A A    
995	. . . . . A A A A A A A A A A A A A A    
996	. . . . . G G G G G G G G G G G G G G    
997	. . . . . C C C C C C C C C C C C C C    
998	. . . . . C C C C C C C C C C C C C C    
999	. . . . . A A A A A A A A A A A A A A    
1000	. . . . . C C C C C C C C C C C C C C    
1001	. . . . . A A A A A A A A A A A A A A    
1002	. . . . . T T T T T T T T T T T T T T    
1003	. . . . . C C C C C C C C C C C C C C    
1004	. . . . . A A A A A A A A A A A A A A    
1005	. . . . . G G G G G G G G G G G G G G    
1006	. . . . . C C C C C C C C C C C C C C    
1007	. . . . . T T T T T T T T T T T T T T    
1008	. . . . . G T C C C C C C C C C C C C    
1009	. . . . . . . G G G G G G G G G G G G    
1010	. . . . . . . T T T T T T T T T T T T    
1011	. . . . . . . A A A A A A A A A A A A    
1012	. . . . . . . G G G G G G G G G G G G    
1013	. . . . . . . G G G G G G G G G G G G    
1014	. . . . . . . T T T T T T T T T T T T    
1015	. . . . . . . T T T T T T T T T T T T    
1016	. . . . . . . C C C C C C C C C C C C    
1017	. . . . . . . A A A A A A A A A A A A    
1018	. . . . . . . T T T T T T T T T T T T    
1019	. . . . . . . T T T T T T T T T T T T    
1020	. . . . . . . A A A A A A A A A A A A    
1021	. . . . . . . T T T T T T T T T T T T    
1022	. . . . . . . G G G G G G G G G G G G    
1023	. . . . . . . A A A A A A A A A A A A    
1024	. . . . . . . T T T T T T T T T T T T    
1025	. . . . . . . T T T T T T T T T T T T    
1026	. . . . . . . C C C C C C C C C C C C    
1027	. . . . . . . G G G G G G G G G G G G    
1028	. . . . . . . A A A A A A A A A A A A    
1029	. . . . . . . C C C C C C C C C C C C    
1030	. . . . . . . A A A A A A A A A A A A    
1031	. . . . . . . G G G G G G G G G G G G    
1032	. . . . . . . T T T T T T T T T T T T    
1033	. . . . . . . A A A A A A A A A A A A    
1034	. . . . . . . C C C C C C C C C C C C    
1035	. . . . . . . T T T T T T T T T T T T    
1036	. . . . . . . T T T T T T T T T T T T    
1037	. . . . . . . G G G G G G G G G G G G    
1038	. . . . . . . T T T T T T T T T T T T    
1039	. . . . . . . T T T T T T T T T T T T    
1040	. . . . . . . T T T T T T T T T T T T    
1041	. . . . . . . C C C T C C C C C C C C    
1042	. . . . . . . T T T . T T T T T T T T    
1043	. . . . . . . G G G . G G G G G G G G    
1044	. . . . . . . C C C . C C C C C C C C    
1045	. . . . . . . A G G . A A A A A A A A    
1046	. . . . . . . G . . . G G G G G G G G    
1047	. . . . . . . T . . . T T T T T T T T    
1048	. . . . . . . A . . . A A A A A A A A    
1049	. . . . . . . T . . . T T T T T T T T    
1050	. . . . . . . G . . . G G G G G G G G    
1051	. . . . . . . A . . . A A A A A A A A    
1052	. . . . . . . A . . . A A A A A A A A    
1053	. . . . . . . C . . . C C C C C C C C    
1054	. . . . . . . T . . . T T T T T T T T    
1055	. . . . . . . T . . . T T T T T T T T    
1056	. . . . . . . T . . . T T T T T T T T    
1057	. . . . . . . T . . . T T T T T T T T    
1058	. . . . . . . A . . . A A A A A A A A    
1059	. . . . . . . A . . . A A A A A A A A    
1060	. . . . . . . A . . . A A A A A A A A    
1061	. . . . . . . G . . . G G G G G G G G    
1062	. . . . . . . T . . . T T T T T T T T    
1063	. . . . . . . T . . . T T T T T T T T    
1064	. . . . . . . A . . . A A A A A A A A    
1065	. . . . . . . A . . . A A A A A A A A    
1066	. . . . . . . T . . . T T T T T T T T    
1067	. . . . . . . A . . . A A A A A A A A    
1068	. . . . . . . A . . . A A A A A A A A    
1069	. . . . . . . A . . . A A A A A A A A    
1070	. . . . . . . A . . . T T T T T T T T    
1071	. . . . . . . . . . . T T T T T T T T    
1072	. . . . . . . . . . . T T T T T T T T    
1073	. . . . . . . . . . . C C C C C C C C    
1074	. . . . . . . . . . . A A A A A A A A    
1075	. . . . . . . . . . . T T T T T T T T    
1076	. . . . . . . . . . . A A A A A A A A    
1077	. . . . . . . . . . . A A A A A A A A    
1078	. . . . . . . . . . . T T T T T T T T    
1079	. . . . . . . . . . . T T T T T T T T    
1080	. . . . . . . . . . . T T T T T T T T    
1081	. . . . . . . . . . . A A A A A A A A    
1082	. . . . . . . . . . . T T C C T T T T    
1083	. . . . . . . . . . . A A . . A A A A    
1084	. . . . . . . . . . . A A . . A A A A    
1085	. . . . . . . . . . . A A . . G A A A    
1086	. . . . . . . . . . . T T . . . T T A    
1087	. . . . . . . . . . . . . . . . . . T    
1088	. . . . . . . . . . . . . . . . . . G    
1089	. . . . . . . . . . . . . . . . . . C    
1090	. . . . . . . . . . . . . . . . . . A    
1091	. . . . . . . . . . . . . . . . . . G    
1092	. . . . . . . . . . . . . . . . . . G    
1093	. . . . . . . . . . . . . . . . . . A    
1094	. . . . . . . . . . . . . . . . . . G    
1095	. . . . . . . . . . . . . . . . . . A    
1096	. . . . . . . . . . . . . . . . . . A    
1097	. . . . . . . . . . . . . . . . . . T    
1098	. . . . . . . . . . . . . . . . . . G    
1099	. . . . . . . . . . . . . . . . . . A    
1100	. . . . . . . . . . . . . . . . . . C    
1101	. . . . . . . . . . . . . . . . . . T    
1102	. . . . . . . . . . . . . . . . . . T    
1103	. . . . . . . . . . . . . . . . . . G    
1104	. . . . . . . . . . . . . . . . . . G    
1105	. . . . . . . . . . . . . . . . . . C    
1106	. . . . . . . . . . . . . . . . . . T    
1107	. . . . . . . . . . . . . . . . . . A    
1108	. . . . . . . . . . . . . . . . . . G    
1109	. . . . . . . . . . . . . . . . . . A    
1110	. . . . . . . . . . . . . . . . . . A    
1111	. . . . . . . . . . . . . . . . . . T    
1112	. . . . . . . . . . . . . . . . . . C    
1113	. . . . . . . . . . . . . . . . . . A    
1114	. . . . . . . . . . . . . . . . . . C    
1115	. . . . . . . . . . . . . . . . . . A    
1116	. . . . . . . . . . . . . . . . . . A    
1117	. . . . . . . . . . . . . . . . . . T    
1118	. . . . . . . . . . . . . . . . . . T    
1119	. . . . . . . . . . . . . . . . . . C    
1120	. . . . . . . . . . . . . . . . . . C    
1121	. . . . . . . . . . . . . . . . . . T    
1122	. . . . . . . . . . . . . . . . . . C    
1123	. . . . . . . . . . . . . . . . . . T    
1124	. . . . . . . . . . . . . . . . . . T    
1125	. . . . . . . . . . . . . . . . . . T    
1126	. . . . . . . . . . . . . . . . . . G    
1127	. . . . . . . . . . . . . . . . . . G    
1128	. . . . . . . . . . . . . . . . . . A    
1129	. . . . . . . . . . . . . . . . . . T    
1130	. . . . . . . . . . . . . . . . . . T    
1131	. . . . . . . . . . . . . . . . . . T    
1132	. . . . . . . . . . . . . . . . . . A    
1133	. . . . . . . . . . . . . . . . . . C    
1134	. . . . . . . . . . . . . . . . . . C    
1135	. . . . . . . . . . . . . . . . . . C    
1136	. . . . . . . . . . . . . . . . . . T    
1137	. . . . . . . . . . . . . . . . . . G    
1138	. . . . . . . . . . . . . . . . . . G    
1139	. . . . . . . . . . . . . . . . . . C    
1140	. . . . . . . . . . . . . . . . . . A    
1141	. . . . . . . . . . . . . . . . . . T    
1142	. . . . . . . . . . . . . . . . . . G    
1143	. . . . . . . . . . . . . . . . . . A
```
